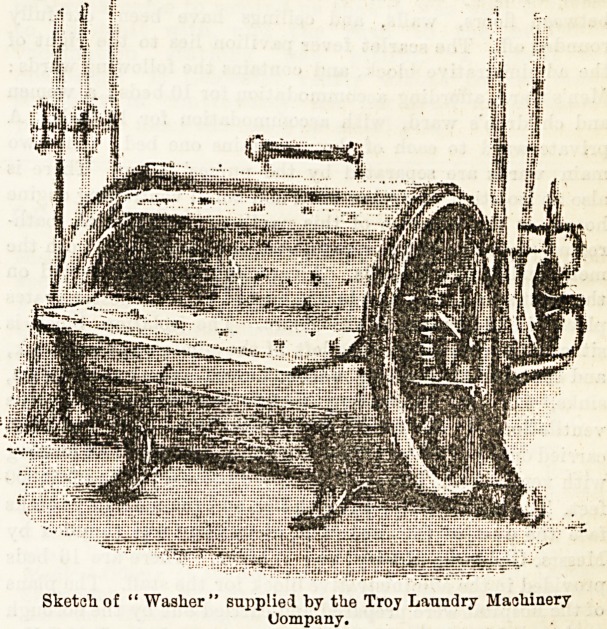# The Hospital Laundry

**Published:** 1894-09-15

**Authors:** 


### PRACTICAL DEPARTMENTS.

### THE HOSPITAL LAUNDRY.

We have treated of this subject before in the pages of The Hospital, but changes so radical and important are being ever made in this department in the way of improved machinery and so forth, that it is well now and again to review the progress made.

The great desirability for every hospital and institution of the kind having its own laundry department is, of course, now widely recognized. It is naturally more economical and better from every point of view that so important a detail of the domestic organization should be under the immediate control of the authorities. As a matter of hygiene, too, it is very imperative that soiled linen shall be cleansed as soon as may be, and that the careful separate washing of linen used for typhoid patients and so on is thoroughly carried out; and this can only be effectively secured when the washing is done "on the premises," under proper supervision.

Machinery has of late years come so largely into use in the laundry that hand-washing is almost superseded, and there can be little doubt, in spite of the old-fashioned prejudice which exists here and there in favour of hand-washing, that a thorough cleansing is better effected with less damage to the materials washed by the modern machines than by the older methods. The initial cost of fitting up such a modern laundry may be considerably greater now than in old days, but the saving in the long run is undeniable.

That this fact is recognised by hospital authorities may be seen by a visit to the laundry of any modern and up-to-date institution. At Guy's, the London, and other large metropolitan hospitals this department forms a distinct feature, and machinery is used to a large extent. A very good example of a modern institutional laundry is that at the Chelsea Infirmary, which, by the kind permission of Miss de Pledge, we visited a few days ago. Here the most modern appliances are in use, but in none of the institution laundries we have visited has hand-labour been so entirely superseded as in the American laundries. In America the cost of labour has no doubt been a powerful factor in the introduction of machinery, whereby the number of hands employed in the large public laundries has been greatly reduced.

The principal object of this present paper is to introduce to our readers a system which prevails largely in America, where machinery is used more than in England for laundry work, even the finest work being well and satisfactorily done by this means.

We recently paid an interesting visit to one of the laundries fitted up on these lines by the Troy Laundry Machinery Company, whose representative in London is Mr. Armstrong, 116, Queen Victoria Street, E.C., and were much impressed by the wondrous contrivances for reducing labour in a laundry to a minimum, and by the thoroughness of all the appliances.

Sketch of "Washer" supplied by the Troy Laundry Machinery Company.

Our first illustration shows a specimen of a hydraulic washer, the machine which first receives the soiled linen. It consists of a double cylinder, both being of sheet brass, the innermost one studded with embossed holes, to prevent any possibility of the edges wearing the linen. The outer cylinder contains the steam coil and the water, an ever fresh supply of the latter being easily regulated by a simple movement. There is an automatic reversing movement (about three revolutions each way) which prevents the contents of the washer becoming tangled or knotted together. Washers of this description are, of course, well known, and similar ones will be found in most modern laundries. English makers, perhaps, more frequently use copper for the inside cylinder, and wood for the outermost.

---

We have been asked to state, in reference to a description of "A Chair for the Nursery," given in The Hospital for August 11th last (p. 399), that Mr. C. Fowle, whose name is there mentioned, is not the manufacturer of the chairs in question, though they may be obtained from him.

## Figures and Tables

**Figure f1:**